# Fibrous Aerogels for Solar Vapor Generation

**DOI:** 10.3389/fchem.2022.843070

**Published:** 2022-02-14

**Authors:** Chengjian Xu, Junyan Zhang, Mina Shahriari-Khalaji, Mengyue Gao, Xiaoxiao Yu, Changhuai Ye, Yanhua Cheng, Meifang Zhu

**Affiliations:** State Key Laboratory for Modification of Chemical Fibers and Polymer Materials, College of Materials Science and Engineering, Donghua University, Shanghai, China

**Keywords:** solar vapor generation, fibrous aerogels, porous structures, thermal management, water management

## Abstract

Solar-driven vapor generation is emerging as an eco-friendly and cost-effective water treatment technology for harvesting solar energy. Aerogels are solid materials with desirable high-performance properties, including low density, low thermal conductivity, and high porosity with a large internal surface, which exhibit outstanding performance in the area of solar vapor generation. Using fibers as building blocks in aerogels could achieve unexpected performance in solar vapor generation due to their entangled fibrous network and high surface area. In this review, based on the fusion of the one-dimensional fibers and three-dimensional porous aerogels, we discuss recent development in fibrous aerogels for solar vapor generation based on building blocks synthesis, photothermal materials selection, pore structures construction and device design. Thermal management and water management of fibrous aerogels are also evaluated to improve evaporation performance. Focusing on materials science and engineering, we overview the key challenges and future research opportunities of fibrous aerogels in both fundamental research and practical application of solar vapor generation technology.

## Introduction

Freshwater consumption has risen in tandem with the world’s growing population. Freshwater scarcity and energy depletion have become global issues that will have an impact on human health, economic development, and, ultimately, social progress (Mekonnen Mesfin and Hoekstra Arjen, 2017). Solar energy and seawater are two seemingly inexhaustible sources of energy on Earth (Lewis Nathan, 2016; Cavusoglu et al., 2017). Water purification using widely distributed solar energy has been viewed as a promising sustainable solution to overcome the problem of freshwater scarcity (Chen C. et al., 2019). In comparison to traditional purification methods such as reverse osmosis (Elimelech and Phillip William, 2011) and multistage flash distillation (Shahzad et al., 2017), which require a lot of energy, clean water purification via solar evaporation could reduce energy consumption and emissions substantially (Deng et al., 2017).

In a typical solar vapor generation (SVG) process, sunlight is absorbed by the light absorber and converted into heat. Meanwhile, water is transported to the surface of evaporator through the water channel, absorbing the heat energy generated by light absorber and evaporate to escape from the system. In this process, the heat utilization efficiency generally could not reach to 100% as partial generated heat is inevitably lost to the surrounding environment via conduction, radiation and convection to the water and environment. To quantitatively evaluate the performance of the absorbers, solar thermal conversion efficiency (*η*) is determined by the percentage of energy utilized to generated vapor in the whole energy input, which is defined as (Zeng et al., 2011; Zhou et al., 2016):

$$\eta =\dot{m}h_{LV}/C_{opt}q_{solar}$$

in which $\dot{m}$ refers to the mass flux of vapor (water evaporation rate), $h_{LV}$ refers to the total evaporation enthalpy change, which includes sensible heat and phase transform from liquid to vapor, $C_{opt}$ represents the optical concentration and $q_{solar}$ is the nominal solar irradiation of 1 kW m^−2^. To increase solar thermal conversion efficiency of SVG evaporator, the mass flux of vapor should be larger. It means the heat energy should be used to generate water vapor dominantly. Therefore, optimized evaporator structure design and construction are necessary.

Three-dimensional (3D) aerogels are novel porous materials with desirable high-performance properties, such as low density, high porosity (>90%) with a large internal surface (>100 m^2^ g^−1^), thermal conductivity (<0.03 W m^−1^ K^−1^) that could be used as the substrate in the SVG system with the function of floating layer and thermal insulation layer (Hu and Zhu, 2019; Yashim et al., 2021).

Fibers, due to their distinguishing characters of high flexibility, large aspect ratio, high specific surface area, and great load-bearing ability (Weng et al., 2020), have been widely used for SVG design over the last decade. Traditional cotton fabric (Wu et al., 2019), carbon fibers (Fang et al., 2019), cellulose nanofibers (Cao et al., 2021), and other one-dimensional (1D) fiber materials were used as the building block of substrate layer or photothermal materials. The combination of fiber materials and 3D aerogels is propitious for SVG: 1) high porosity with directional rough pore structure augments light absorption, reducing reflection and enhancing scattering (Qiu et al., 2019); 2) low thermal conductivity limits thermal loss to the bulk water (Wicklein et al., 2014); 3) directional connected pores inside aerogels serve as channels to transport water for adequate water supply during evaporation process (Jiang et al., 2018). Benefitting from these advantages, the 3D fibrous aerogels are becoming increasingly popular among various choices by combining thermal management and water management to improve SVG performance.

**FIGURE 1:**
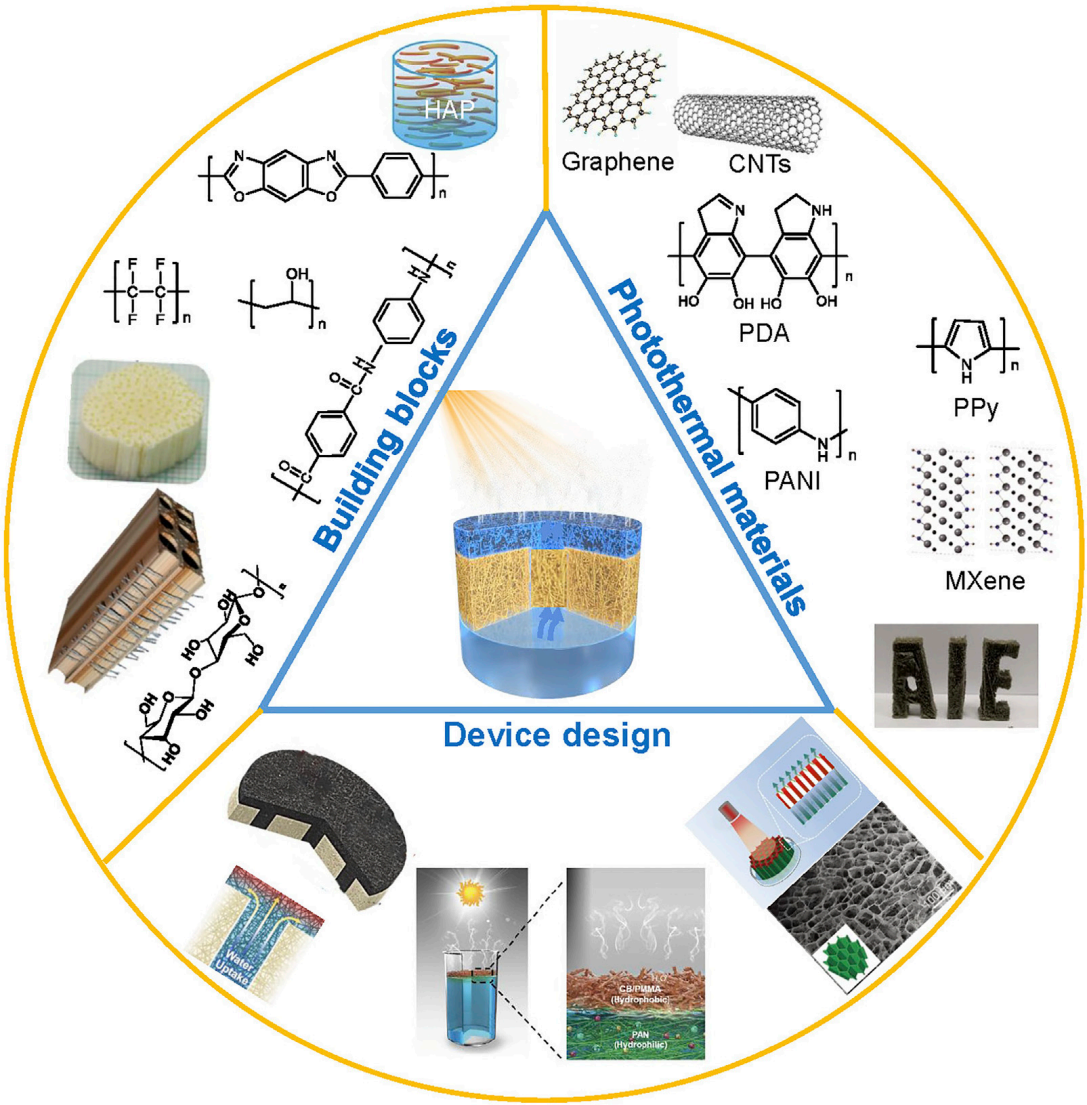
Schematic showing the key sections of fibrous aerogels fabrication for solar vapor generation described in this review. Building blocks synthesis, photothermal materials selection, porous structures construction and devices design are reviewed. Reprinted with permission from (Zhang Q. et al., 2020). Copyright (2020) American Chemical Society. Reprinted with permission from (Li J. et al., 2020). Copyright (2020) American Chemical Society. Reprinted with permission from (Zhang et al., 2018). Copyright (2018) American Chemical Society. Reprinted with permission from (Gao T. et al., 2019). Copyright (2019) WILEY VCH. Reprinted with permission from (Xu et al., 2018). Copyright (2018) WILEY VCH. Reprinted with permission from (Xiong et al., 2021). Copyright (2021) WILEY VCH. Reprinted with permission from (Li et al., 2020a). Copyright (2020) American Chemical Society. Reprinted with permission from (Naguib et al., 2011). Copyright (2011) WILEY VCH.

**FIGURE 2:**
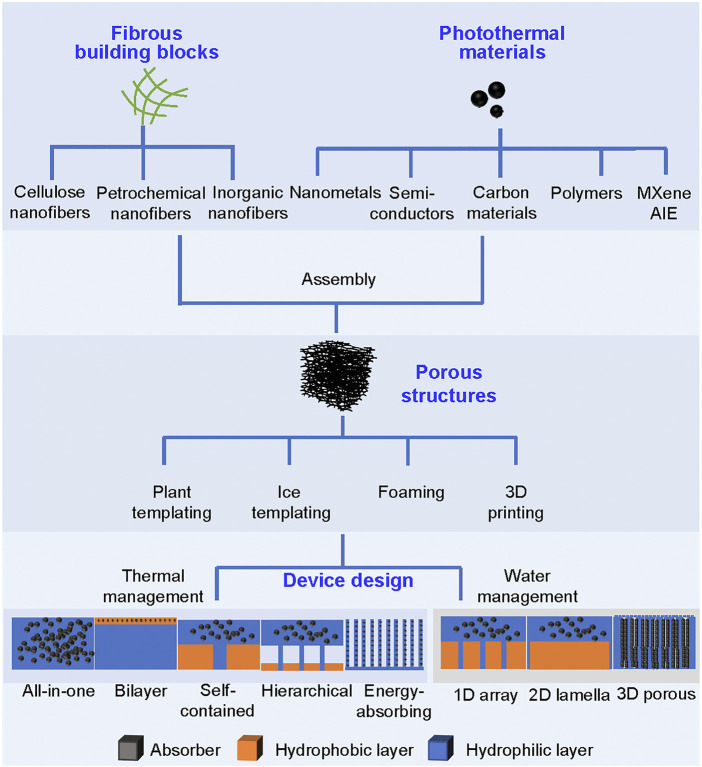
Preparation principles of fibrous aerogels for solar vapor generation.

Herein, we review recent progress in the advancement of SVG based on 3D fibrous aerogels (Figure 1) in terms of 1) the synthesis of fiber materials as building blocks for constructing aerogels with robust mechanical stability; 2) the selection of photothermal materials with broadband absorption and good interfacial binding ability with aerogels to ensure the high solar energy conversion efficiency and long-term durability; and 3) the device design strategy for fibrous aerogels with optimized thermal and water management (Figure 2). In addition, the key challenges and future research opportunities associated with fibrous aerogels would be also discussed. This review aims to provide a better understanding of fibrous aerogels as an efficient SVG system and its potential applications in conjunction with other fields of energy and environment.

## Fibrous Building Blocks Synthesis

The aerogels composed of fibrous building blocks have robust structure and excellent mechanical performance due to the entanglement of fiber network (Zhang et al., 2021b), which is advantageous for the long-term use of SVG evaporators. The innovation in materials science of fibrous building blocks based on different materials with advanced technology has significantly improved the performance of fibrous aerogels over the last few decades. This section focuses on the selection of the material and preparing methods of fibrous building blocks to fabricate aerogels used in SVG system, mainly including cellulose, petrochemical polymer, and inorganic nanofibers.

### Cellulose Nanofibers

Cellulose materials, which are widely distributed among various types of plants, have been recognized as the popular building blocks to prepare biocompatible and sustainable fibrous aerogels over the last decade(Zhang J. et al., 2019; Zhang et al., 2021b). The high crystallinity of cellulose materials ensures the high strength of aerogels, and the functional groups on the surface are conducive to the strong binding with photothermal materials (Cao et al., 2021). Moreover, the hydrophilic nature of cellulose facilitates the water supply through aerogel with sufficient water channels, and the porous structure created by cellulose fibers ensures the low thermal conductivity to minimize energy loss (Li N. et al., 2021). Combined with these characteristics, cellulose materials are one of the attractive building blocks for SVG systems.

Cellulose can be extracted from a variety of sources, including wood, cotton, bamboo, vegetables, as it is the primary constituent of cell wall, or produced by bacteria via fermentation (Li T. et al., 2021). The cellulose nanofibers separated from cellulose demonstrates to be outstanding materials for constructing SVG. Nanocellulose is classified into three types based on synthesis technique and conditions: cellulose nanocrystals (CNC), cellulose nanofibers (CNF), and bacterial nanocellulose (BNC) (Kang et al., 2017; Kim et al., 2019; Zhang et al., 2021b). CNCs are highly crystalline (rod-like or rice-husk like morphology) and have a low aspect ratio of 5–30 with diameter of 5–20 nm and length of 50–350 nm (Foster et al., 2018). For CNCs with low aspect ratio nature, they are normally used as filler for nanocomposite materials, which are not applicable for aerogel construction as SVG evaporator. CNFs have high aspect ratio with diameters of tens of nanometer and length of micrometer range (Chen et al., 2021c). Using CNFs to construct aerogels, the entangled fibrous network endows final aerogels with lower shrinkage rate (<7%) and high modulus (as high as 5.93 MPa). BNCs have a polymerization degree of up to 8,000 and a crystallinity of over 70% with diameter less than 100 nm. Compared with CNFs, BNCs show higher elastic modulus and mechanical robustness (Esa et al., 2014). The resulted aerogels by using BNCs as building blocks exhibit the highest modulus mechanical property (as high as 16.7 MPa) among cellulose aerogels. Meanwhile, high porosity and high specific surface area of BNCs aerogels facilitate good thermal insulation and water transportation. However, the problem faced by BNCs are that the large-scale production of BNCs is difficult, and commercialization remains questionable. Therefore, it is crucial to use appropriate raw materials to prepare cellulose aerogels, as well as to understand the process of nanocellulose synthesis.

**FIGURE 3:**
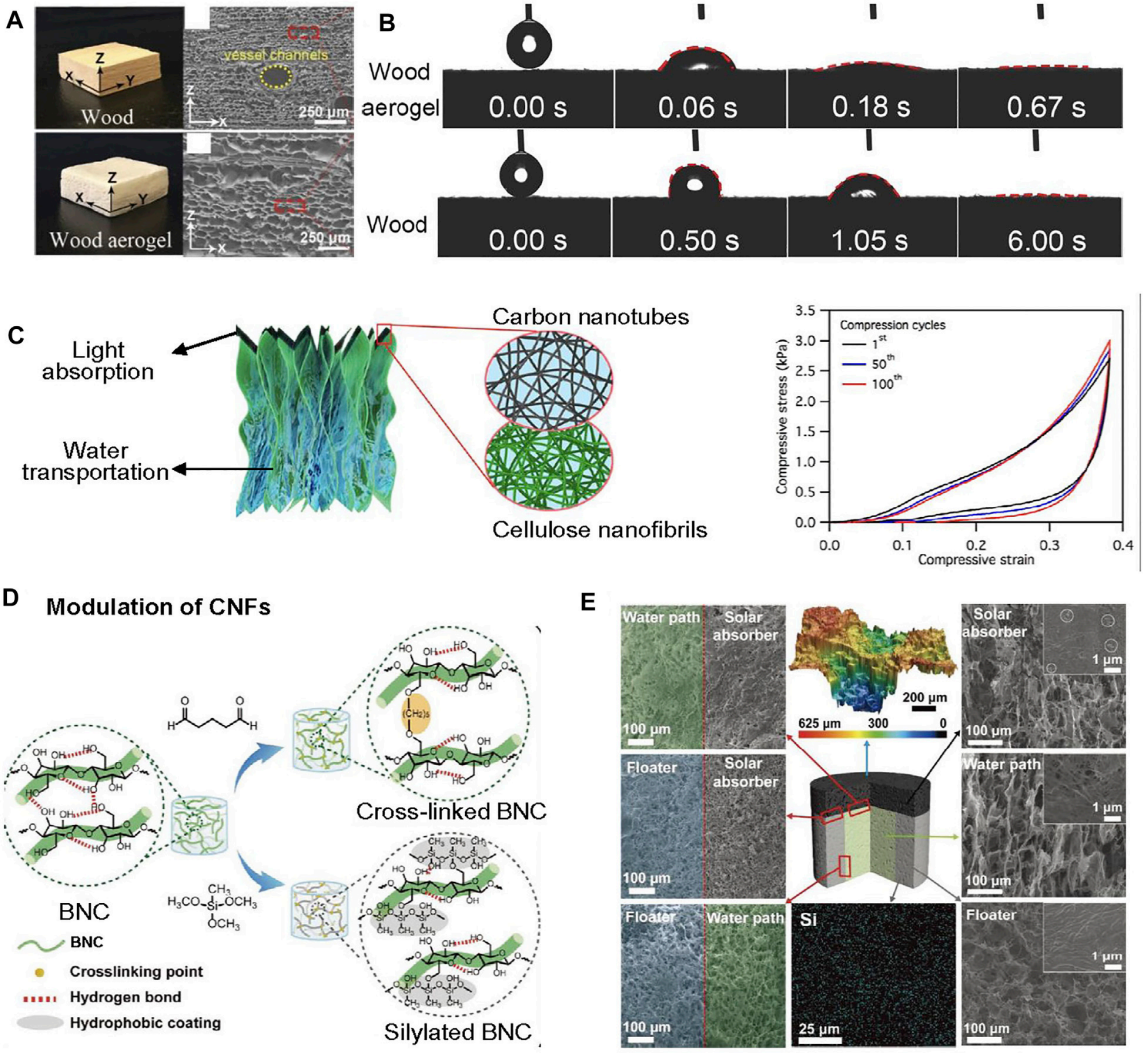
Cellulose nanofibers as building blocks for fibrous aerogels. **(A)** The photographs and corresponding SEM images of the natural balsa wood and the wood-derived aerogel. **(B)** Water contact angle images of wood and wood-derived aerogels in **(A)** when a water droplet was dropped on their surface, indicating better hydrophilicity of the wood-derived aerogels compared with that of the original wood. Reprinted with permission from (Zhang Q. et al., 2020). Copyright (2020) American Chemical Society. **(C)** (left) Schematic illustration of the bilayer CNF−CNTs aerogel used for solar steam generation. The SVG device is composed of a bilayer structure of a bulk CNF aerogel with CNTs depositing on the surface. (right) The compressive cyclic test of CNF-CNTs aerogel in water demonstrates the mechanical robustness of aerogel. Reprinted with permission from (Jiang et al., 2018). Copyright (2018) American Chemical Society. **(D)** Synthetic routes and chemical structures of the bilayer aerogel. **(E)** 3D optical microscope images and cross-sectional SEM images of each component in the bilayer aerogel. Reprinted with permission from (Li N. et al., 2021). Copyright (2020) WILEY VCH.

Plant cell walls are a hierarchical structure of cellulose macromolecules bonded with lignin and hemicellulose, which is widely used as cellulose resources through chemical or physical treatment (Filson and Dawson-Andoh, 2009). The bonded structure forms fibrous packets known as microfibrils, which are composed of crystalline and amorphous regions (Lu and Hsieh, 2012). The amorphous region is dissolved after chemical hydrolysis, allowing longitudinal cellulose nanofibers to be released. For example, using chemical hydrolysis with the mixed solution of sodium hydroxide (NaOH) and sodium sulfite (Na_2_SO_3_) to treat wood (a source of cellulose) for fabricating wood-based fibrous aerogel by partially removing lignin and hemicellulose. Compared to natural wood, the lamellar structure of fibrous wood aerogel is distinct, endowing wood aerogel with low thermal conductivity (0.0418 W m^−1^·K^−1^) (Figure 3A). Meanwhile, the hydrophilicity of wood aerogel was enhanced after treatment, which facilitated water transportation through the aerogel and improved desalination performance simultaneously (Figure 3B). Due to the higher water wettability of the wood aerogel, the evaporator exhibited salt-rejection behavior with efficient water and salt ion transport ability during 120 h desalination, demonstrating its high performance for SVG (Zhang Q. et al., 2020).

Different from above subtractive modifications to fabricate wood aerogel, the exfoliation of CNFs from plant cell walls requires significant amount of energy during the chemical separation. CNFs face the problems such as the dispersion and functionality, which are typically limited due to the hydrogen bonding among nanofibers, the high cost and low efficiency hinder the large-scale production of CNFs (Wang et al., 2016).

Hence, the pretreatment methods like enzymatic hydrolysis to ease the mechanical disintegration process or post-processing of nanofibers to obtain homogeneous nanofiber dispersion are prerequisite for enhancing product performance (Nasir et al., 2017). The hydroxyl group can be modified with various functional groups, and chemical modification can substitute specific reactive groups to the cellulose surface (Luo et al., 2018). In particular, TEMPO (2,2,6,6-tetramethylpiperidine-1-oxyl radical)-oxidization is mostly used for cellulose nanofibers surface decoration, but without altering the original crystallinity of cellulose (Isogai et al., 2011). That is, the fiber length is preserved to the greatest extent. TEMPO-mediated oxidation converts the C6 primary alcohol of cellulose into carboxylic acid (Tang et al., 2017). Owing to the electrostatic repulsion between cellulose microfibrils, the complete dispersion of cellulose nanofibers with gentle disintegration can be achieved. Softwood was used to obtain CNFs with a width of approximately 3–5 nm and a length of several hundreds of nanometers. Following freeze-drying, the CNF aerogel was coated with photothermal materials of carbon nanotubes (CNTs) to acquire a bilayer structure (Figure 3C, left). Due to the long fiber length and the entangled network, the obtained CNF aerogel showed good mechanical robustness, which exhibited stability during the compression−recovery cycle test without breaking apart in water (Figure 3C, right) (Jiang et al., 2018).

Unlike the mechanical or chemical treatment of cellulose extracted from plants, the nanocellulose directly produced by certain aerobic bacterial species called bacterial nanocellulose (BNC) and possesses high crystallinity (up to 90%) and fewer impurities (Yamanaka et al., 1989; Shahriari-Khalaji et al., 2020). With adequate sugar and oxygen in the medium for fermentation, BNC is generated in forms of 3D interconnected nanofibrous network with a diameter ranging from 20 to 100 nm. The mechanical stability and flexibility of BNC are notable due to its high crystallinity and polymerization (Seddiqi et al., 2021). Furthermore, the high surface area and abundant hydroxyl groups in BNC provide enough sites for photothermal materials to load with stable interface contact, making it an excellent building block choice for SVG (Cao et al., 2021; Zhang et al., 2021c).

Recently, the all-cellulose aerogel for vapor generation using BC-based nanofibers as building blocks was developed (Li N. et al., 2021). Glutaraldehyde and silane were respectively used to crosslink with BNC nanofibers to afford the resulted aerogel with hydrophilic and hydrophobic property (Figure 3D). The resulted glutaraldehyde cross-linked BNC aerogel was used for photothermal layer and water transport, while silylated hydrophobic BNC aerogel was applied for the floating layer to reduce the energy loss to bulk water. Such all-fibrous aerogel is beneficial for mechanical stability for long term use with entangled fibrous network (Figure 3E). The solar test over 9 h of aerogel under one-sun with 1.81 kg m^−2^ h^−1^ evaporation rate demonstrated the long-term stability of cellulose aerogel.

The main problem of BNCs is the difficulty in the large-scale production and commercialization. Therefore, the regenerated cellulose nanofibers are another choice for preparing SVG. Various starting materials including cotton and cellulose acetate can be reproduced to generate cellulose nanofiber through dissolution-regeneration and solvent exchange process. (Teixeira et al., 2020). Alkali (NaOH or LiOH) solution systems, alkali/water/urea or thiourea, N-Methylmorpholine-N-Oxide (NMMO)/water and ion liquids (ILs) are usually used to dissolve cellulose (Long et al., 2018). Using the solution as spinning solution, the cellulose nanofibers could be prepared (Frey, 2008; Maharjan et al., 2021). The NMMO/water system is a relatively simple, resource preserving and environmentally friendly method to produce regenerated cellulose fibers (Fink et al., 2001). The N-O dipoles in NMMO form hydrogen bonds with the hydroxyl group of cellulose, thus breaking the intrinsic hydrogen bonding linkage of cellulose to achieve cellulose dissolution. Though electrospinning at 70–110°C during the spinning process from NMMO/water, the cellulose nanofibers are produced with a diameter of 250–750 nm, and the degree of crystallinity of cellulose fibers could be controlled by the spinning parameters (Kim et al., 2006). The cellulose acetate is applicable for electrospinning in the solvent system of dimethylacetamide (DMAC) and acetone. Coupled with silica sol, the cellulose nanofiber film was fabricated through electrospinning. (Pirzada et al., 2020). These nanofibers are potential building blocks for SVG aerogel construction.

### Petrochemical Polymer Nanofibers

In addition to natural polymer fibers, using petrochemical polymer nanofibers as building block, such as poly (paraphenylene terephthalamide) (PPTA), poly(p-phenylenebenzobisoxazole) (PBO), and polyacrylonitrile (PAN), to prepare aerogels for SVG system is also appealing because of their additional functions (e.g., mechanical robustness, high temperature stability, chemical resistance).

**FIGURE 4:**
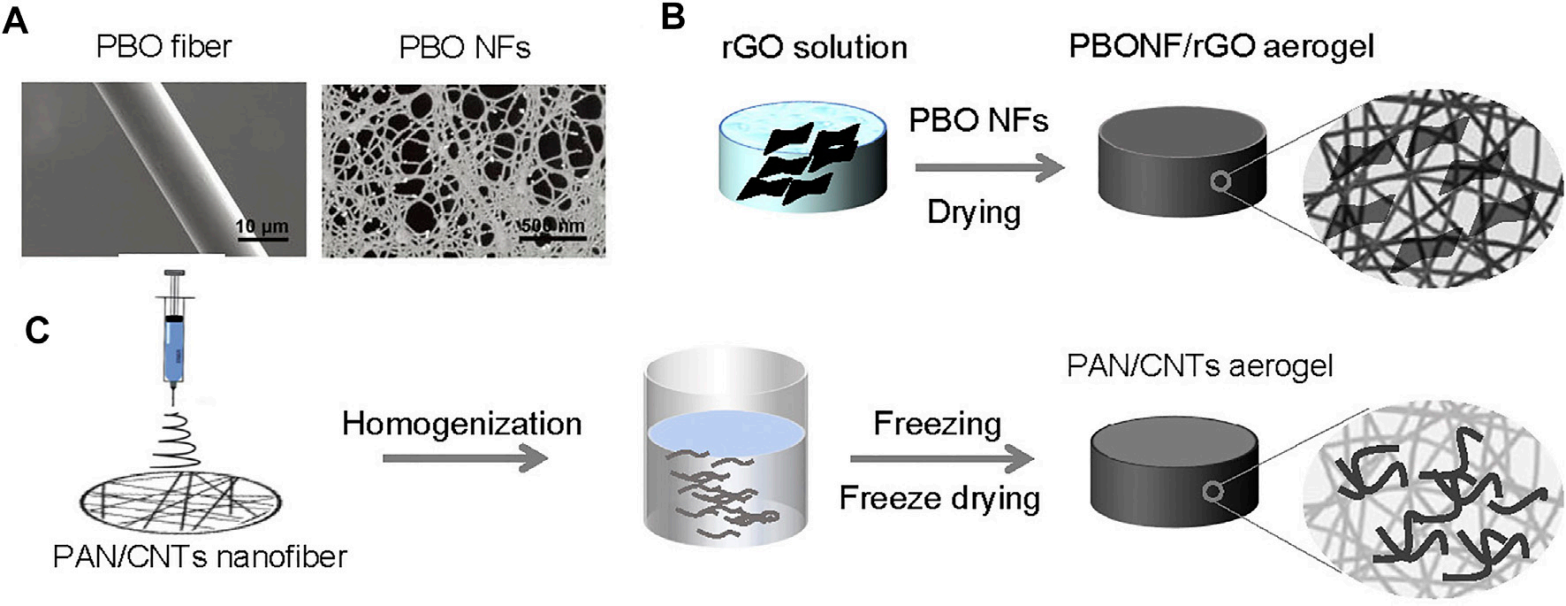
Petrochemical polymer nanofibers as building blocks for fibrous aerogels. **(A)** SEM images of the commercial PBO fiber and the exfoliated PBO nanofibers. Reprinted with permission from (Chen et al., 2018). Copyright (2018) The Royal Society of Chemistry. **(B)** Synthesis of nanofibrous SVG (all-in-one structure) using PBO nanofibers as building blocks and rGO as photothermal materials(Chen M. et al., 2021). **(C)** Synthesis of CNTs-containing PAN nanofibers and corresponding SVG aerogels (all-in-one structure) (Liu et al., 2022).

PBO nanofibers are developed using chemical treatment to exfoliate microfibers into nanofibers. The general process is to split the strong bonding interactions inside fibers such as hydrogen bonding and van der Waals force, by using acid, alkali, and organic solvent with a downsizing process (Uchida and Furukawa, 2014; Ifuku et al., 2015). Compared to PBO microfiber, PBO nanofiber is beneficial for the transportation of water and relaxation of vapor because of its large surface area (Figure 4A; Chen et al., 2018). It is worth noting that PBO nanofiber itself can transfer light to heat. Cooperating with photothermal materials of reduced graphene oxide, the solar-vapor conversion efficiency reached 98.4% under one-sun illumination stably due to the excellent mechanical and thermal properties of PBO nanofibers (Figure 4B; Chen M. et al., 2021).

PAN nanofibers have good weather and solar resistance due to the highly polar acrylonitrile groups on the polymer chain to afford strong intermolecular interactions (Gupta and Afshari, 2009). With such properties, the fibrous aerogel comprising PAN nanofibers as a well substrate is demonstrated to show environmental stability (Figure 4C). With hierarchical pore structure cooperated with CNTs as solar absorber, the composite aerogel outperformed most nanofibrous membranes with a fast evaporation rate of 2.13 kg m^−2^ h^−1^ and high solar-vapor conversion efficiency of 94.5% under one-sun (Liu et al., 2022).

With chemical treatment or electrospinning method, the high-performance polymer nanofiber could be produced with the expected properties used for SVG building block. However, the chemical solvent used during the process and time-consuming procedure are not beneficial for commercial utilization. The polymer nanofiber preparation can be simplified if the melt spinning can be used with specific deign, such as ultrathin spinneret and fiber cross-section structure design corresponding to the spinneret (Karaca and Ozcelik, 2007; Kara et al., 2012).

### Inorganic Nanofibers

Inorganic materials are another important materials that are widely used in our daily life because of their stability, corrosion, and high-temperature resistance (Ziegler et al., 2017). Being highly chemically stable, the inorganic materials are suitable to be used as evaporator building blocks in harsh conditions such as extremely saline water, polluted waste water, and so on.

**FIGURE 5:**
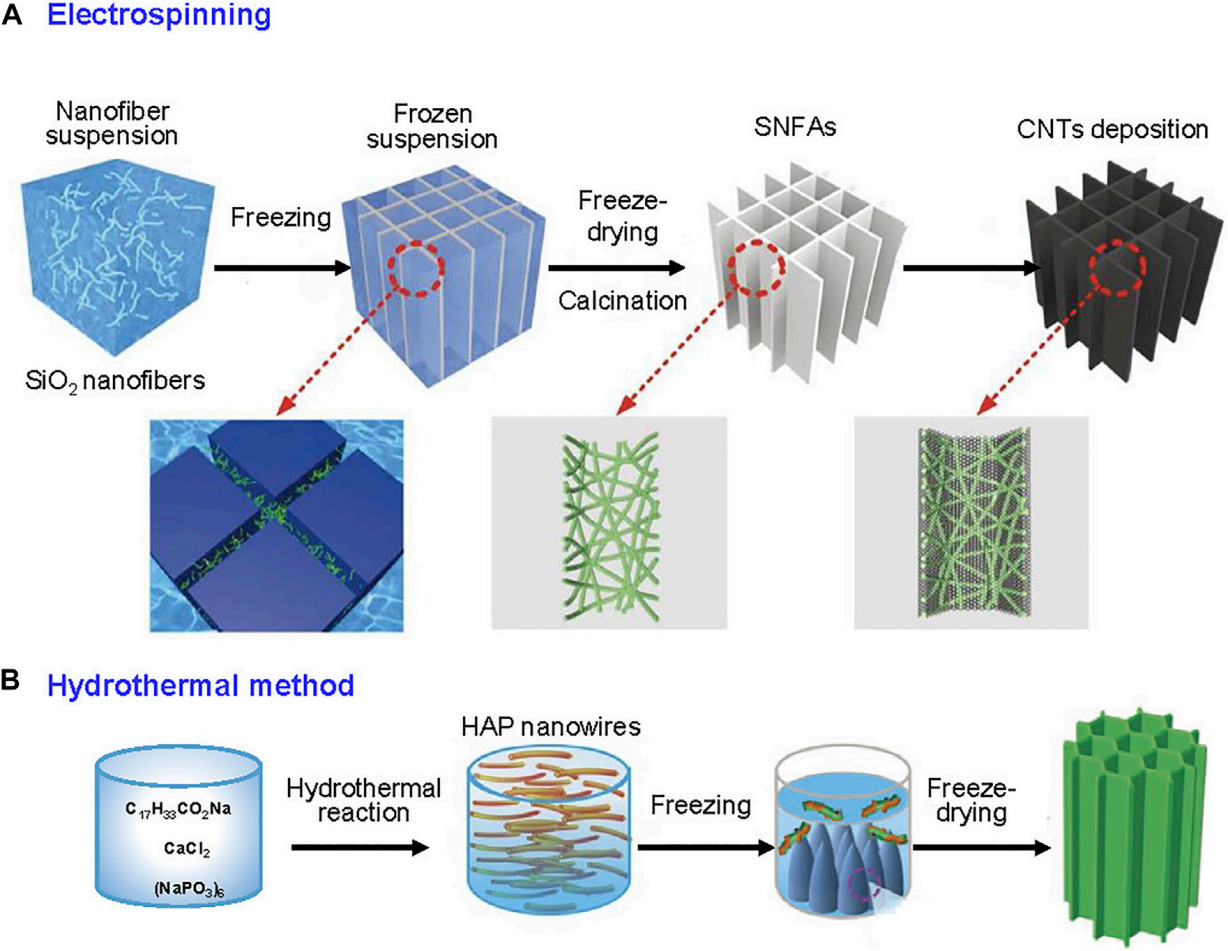
Inorganic nanofibers as building blocks for fibrous aerogels. **(A)** Fabrication principles of the SiO_2_ nanofibrous aerogel. Reprinted with permission from (Dong et al., 2020). Copyright (2020) WILEY VCH. **(B)** Fabrication process of HAP nanowires and their corresponding aerogels. Reprinted with permission from (Zhang et al., 2018). Copyright (2018) American Chemical Society. Reprinted with permission from (Xiong et al., 2021). Copyright (2021) WILEY VCH.

Similar to polymer materials, electrospinning is the popular strategy to prepare inorganic nanofiber among various methods to fabricate fibrous inorganic building blocks. Silicon dioxide (SiO_2_) nanofiber aerogel was first been fabricated by electrospun nanofiber (Si et al., 2014). The precursor tetraethyl orthosilicate (TEOS) was hydrolyzed and added into polyvinyl alcohol (PVA) solution prior to electrospinning at a suitable voltage and rate. Then, PVA was removed after high-temperature calcination, yielding pure SiO_2_ fibrous membrane. The SiO_2_ nanofiber aerogel was constructed by homogenizing of nanofiber membrane to obtain dispersion and then freeze-drying. With an inspiration of reed leaves, the above SiO_2_ fibrous aerogel with parallel-arranged vasculature was chosen as substrate and polypyrrole or CNTs deposited on the substrate as solar absorber (Figure 5A; Dong et al., 2020; Dong et al., 2021). The salt resistance of aerogel is outstanding, which could work stably in high concentration brine.

Aluminum oxide/titanium dioxide (Al_2_O_3_/TiO_2_) inorganic nanofiber through electrospun method is also been adopted to prepare fibrous aerogel for SVG (Meng et al., 2020). A homogeneous precursor containing titanium isopropoxide (TTIP) and aluminum acetylacetonate (Al(acac)_3_) to obtain Al_2_O_3_/TiO_2_ nanofibers. Benefitting from the photocatalytic property of TiO_2_, the developed Al_2_O_3_/TiO_2_ fibrous aerogel also exhibited surprisingly photodegradation property with 91.3% removal ratio of polluted wastewater.

Hydroxyapatite (HAP) is a mineral predominantly found in our bones and teeth (Farokhi et al., 2018). Ultralong flexible HAP nanowires could be prepared by hydrothermal method and used as substrate for SVG (Figure 5B; Xiong et al., 2021). HAP nanowires have diameters of 10–20 nm and lengths of hundred micrometers. Through a unidirectional freeze-drying approach, the HAP nanowires self-assembled into aerogel with vertically aligned channels. The mechanical properties of aerogel were outstandingly attributed to the orientated structure, with no structural deformation after pressing tests. Moreover, the water could flow quickly through aligned channels to ensure adequate water supply. Due to the abundant surface functional groups of HAP nanofibers, they showed good affinity with polymers, such as antibacterial-active chitosan. Besides high SVG efficiency (86.7%), the HAP-based aerogel also manifested the bacteria-disinfected capability with 100% removal efficiency of both *E. coli* and *S. aureus* after filtration treatment.

Compared with petrochemical polymer nanofibers, the inorganic nanofibers characterized with chemical stability, anti-corrosion property, and high-temperature resistance, are preferred for long-term durability of SVG. The question of inorganic nanofibers is that how to simplify the preparation process. The bottom-to-top concept is a classic method. The polymerization method of inorganic oligomer is promising to produce high-performance inorganic nanofiber, which is a worthy reference(Liu Z. et al., 2019).

## Photothermal Materials Selection

Photothermal materials (as solar absorbers) are another important component in solar vapor generator with solar-to-heat function to give rise to water vapor (Karami et al., 2021). The solar spectrum ranges from 280 to 2,500 nm, with visible and infrared (IR) light contributing the most of energy (97%) (Gueymard, 2004; Liu F. et al., 2019). High light absorption across the entire solar spectrum and high light-to-heat conversion efficiency are crucial for photothermal materials. Nanotechnology has enabled the remarkable development of photothermal materials over the last decade. So far, plasmonic nanometals (Wu et al., 2019; Zhang D. et al., 2021), semiconductors (Ghim et al., 2018), carbonaceous materials (Jiang et al., 2016), polymers(Zou et al., 2021), and their hybrids have been used for capturing solar energy based on fibrous aerogel substrates.

### Plasmonic Nanometals

**FIGURE 6:**
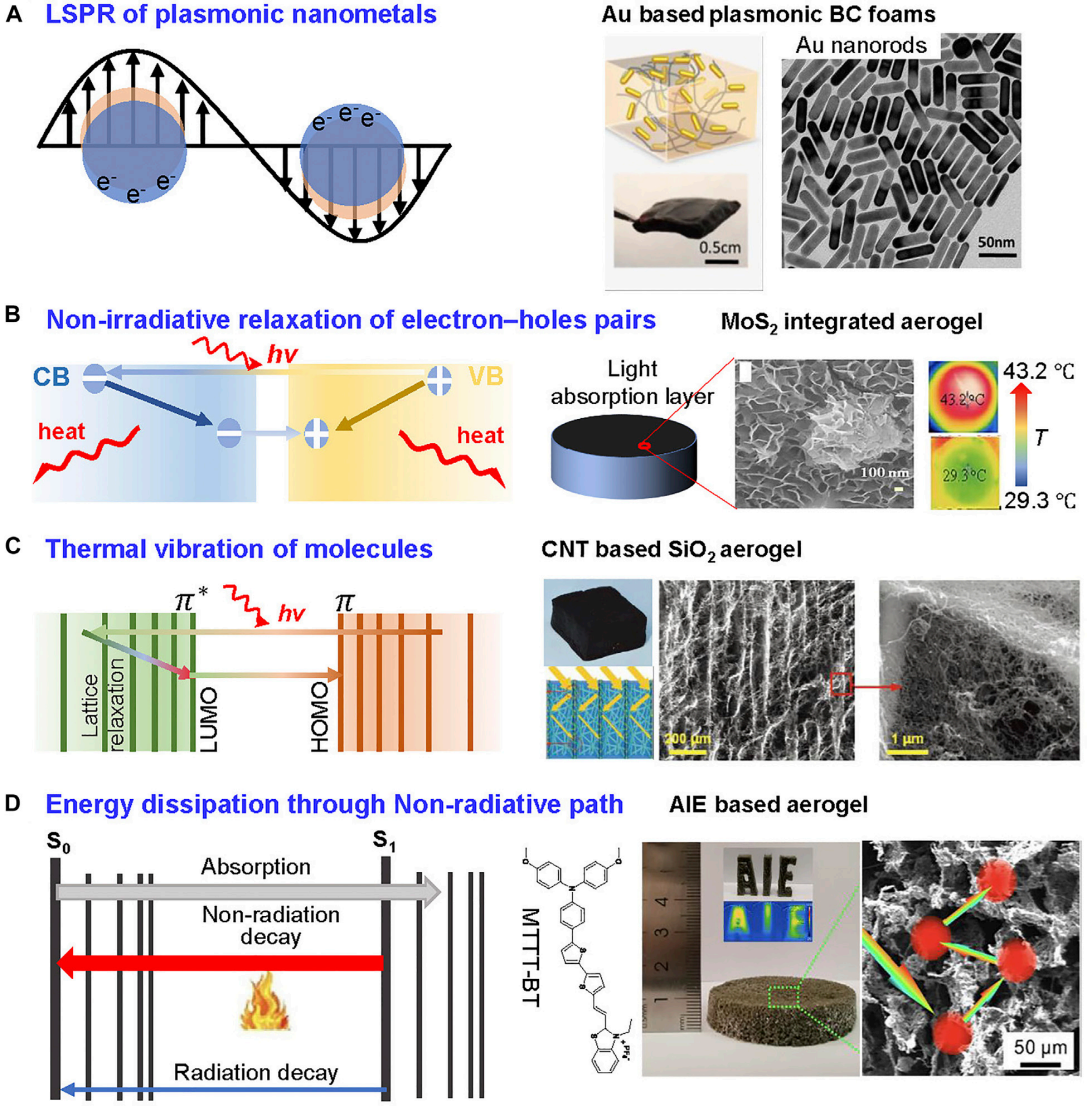
Photothermal mechanisms of various types of photothermal materials and their corresponding demonstrations. **(A)** (left) LSPR effect of plasmonic nanometals (Wang et al., 2019). (right) Plasmonic materials to maximize solar radiation absorption made from gold nanorods and their application as photothermal materials in SVG. Reprinted with permission from (Tian et al., 2016). Copyright (2016) American Chemical Society. **(B)** (left) Non-irradiative relaxation of electron–holes pairs of semiconductor (Chen C. et al., 2019). (right) MoS_2_ nanomaterials as a demonstration for solar evaporator. Reprinted with permission from (Guo et al., 2018). Copyright (2018) American Chemical Society. **(C)** (left) Thermal vibration of carbonaceous and polymer materials (Chen C. et al., 2019). (right) Incorporation of CNTs as photothermal materials into SiO_2_ aerogel with over 98% light extinction efficiency. Reprinted with permission from (Dong et al., 2020). Copyright (2020) WILEY VCH. **(D)** (left) Photothermal mechanism of AIE molecules via non-radiative path (Li et al., 2020b). (right) Chemical structure of the AIE molecule, optical photograph and SEM image of AIE-active nanofibrous aerogel. Reprinted with permission from (Li et al., 2020a). Copyright (2020) American Chemical Society.

Plasmon resonance-based metal nanoparticles have robust light absorption and light-to-heat conversion ability. Localized surface plasmons can be excited by light irradiation of metal nanoparticles. Collective excitation of electrons is triggered when the frequencies of light irradiation and localized surface plasmon resonance (LSPR) of metal nanoparticles match the frequency of light irradiation (Figure 6A, left). As LSPR decays through non-radiative route, excited electrons with high kinetic energy may be converted to heat via a joule mechanism (Brongersma et al., 2015; Liu G. et al., 2017). Various plasmonic nanometals, including Au, Ag and Cu, have been coupled with fibrous substrates used for SVG (Wu et al., 2019; Zhang D. et al., 2021).

Fibrous aerogels could efficiently load plasma nanometals due to their high surface area and abundant surface functional groups, e.g., cellulose. The gold nanorods is a typical plasma material. The SVG evaporator can be obtained by introducing gold nanorods into BNC foam through van der Waals forces and electrostatic interaction (Tian et al., 2016; Figure 6A, right). The plasmonic foam exhibited a significant temperature rise (from 25 to 48°C) under laser irradiation due to high doping ratio of gold nanorods in foam. Besides gold nanorods, copper sulfide is also commonly used as a photothermal material for SVG evaporator. The evaporator aerogels were fabricated by *in-situ* growth of copper sulfide within BNC matrix. The light absorption of the copper sulfide-incorporated evaporator ranges from 97.9 to 100% between 250 and 2,100 nm, enabling an excellent light-to-heat ability. Under one-sun illumination, the surface temperature of dried aerogels could even reach 84.2 C within 30 min, demonstrating the outstanding solar-to-heat ability.

Gogotsi’s group at Drexel University discovered MXene in 2011 through selective etching of the Aluminum(Al) layer of the ternary layered carbide Ti_3_AlC_2_ with hydrofluoric acid (Naguib et al., 2011). MXene, Ti_3_C_2_T_x_ (T = F, O, or OH) has become the popular choice for SVG with excellent photothermal performance over the last decade. Several works have reported that localized surface plasmon resonance effect dominated the light-to-heat process of MXene, and MXene also demonstrated great potential for application in SVG system (Li R. et al., 2017; Lin et al., 2017).

Fibrous aerogel comprising MXene and cellulose nanofibrils showed 95.8% light absorption (200–2,500 nm), and the surface temperature could increase to 70°C in 2 min (Han et al., 2021). The outstanding photothermal property was achieved by combining the inherent photothermal property of MXene and a logically designed fibrous microstructure to improve the multiple scattering and reflection.

### Semiconductors

Semiconducting materials is another category of photothermal agents because of their tunable energy band and solar-to-heat efficiency. The bandgap energy of a semiconductor determines its ability to convert light to heat. In narrow bandgap semiconductors, as the energy of most photons from the incident solar light is higher than that of the bandgap, electron-hole pairs will be generated (Wang et al., 2017). These electron-pairs could follow a non-radiative route and relax to band edges, converting extra energy to heat (Figure 6B, left). Semiconductors with narrow bandgaps, such as molybdenum(IV) sulfide (MoS_2_), dititanium trioxide (Ti_2_O_3_), and ferroferric oxide (Fe_3_O_4_) have been extensively investigated for solar steam generation (Chen et al., 2017; Wang et al., 2017).

In contrast to titanium dioxide (TiO_2_), a traditional semiconductor with a large bandgap (≈3 eV) that can absorb UV light at wavelengths lower than 400 nm, Ti_2_O_3_ could absorb solar energy across the full spectrum, corresponding to a narrow bandgap (≈0.1 eV) and black appearance (Wang et al., 2017). The nanosized Ti_2_O_3_ could further improve the absorption by enhancing light scattering. The light absorption of Ti_2_O_3_ over the whole spectrum could reach 92.5%. It is demonstrated that the surface temperature of device could rise to 50°C at five-sun illumination for 15 min by depositing Ti_2_O_3_ nanoparticles on cellulose membrane. Another example is MoS_2_ nanosheet-incorporated BNC aerogel, which was fabricated via *in-situ* growth to prevent MoS_2_ leakage during application (Ghim et al., 2018). In comparison to pure BNC aerogel exhibiting ∼10% light extinction, the MoS_2_/BNC aerogel demonstrated higher percentage of light extinction (96%). The resulted MoS_2_/BNC aerogel achieved solar evaporation efficiencies of 81.4 and 75.7% under 5.35 kW/m^2^ and 0.76 kW/m^2^ light irradiation, respectively. Furthermore, MoS_2_-integrated aerogel was also demonstrated to show broadband light absorption (>90%) within 200–2000 nm wavelength. After coupling with thermal insulation layer, the resulted SVG showed surface temperature rise to 43.2°C in 5 min under 1 kW/m^2^ light irradiation (Figure 6B, right) (Guo et al., 2018).

The solar-to-heat performance of plasmonic nanometals and semiconductors are outstanding. However, the cost of above-mentioned materials is comparatively high and not suitable for commercial use. Therefore, the lower cost photothermal materials including carbonaceous materials and polymer materials are discussed in the following parts.

### Carbonaceous Materials

The photothermal property of carbonaceous materials stems from lattice vibrations upon light illumination (Chen C. et al., 2019). With a small amount of energy, the loosely held electrons in carbonaceous materials could be excited from π orbital to π* orbital. When an electron relaxes to its ground state, the released energy is converted to heat (Figure 6C, left). In this regard, diverse carbonaceous materials have been investigated, such as graphite, carbon nanotubes (CNTs), graphene oxide (GO)/reduced graphene oxide (rGO), and carbon black (Vélez-Cordero and Hernández-Cordero, 2015; Gao M. et al., 2019).

Graphene is two-dimensional materials that composed of sp^2^ hybridization of carbon atoms, which is a miraculous material in the new century (Geim and Novoselov, 2007; Yu G.-H. et al., 2019). It has been widely employed as the photothermal material for SVG with various structures. In order to achieve the strong interfacial interaction between graphene and fibrous substrate, graphene oxideare usually used as starting materials due to their hydrophilicity, after chemical reduction, reduced graphene oxide were formed *in situ* (Ren et al., 2017; Wang H. et al., 2020). GO nanosheets can be incorporated into the fibrous network *in situ* during the growth of BNC network (Jiang et al., 2016). Compared to pure BC foam, the rGO/BNC aerogel exhibited a large optical extinction (∼96%) with the benefits of rGO nanosheets and nanoscale cellulose fibers. With this design, the evaporator exhibited stable performance and the evaporation rate reached 11.8 kg m^−2^ h^−1^ under ten-sun.

CNTs are another popular carbonaceous material that have been widely used as photothermal material because of 1D morphology working in tandem with fibrous building blocks to achieve high absorption coefficient (Jiang et al., 2018). The CNTs can wrap on the fibrous network of inorganic aerogel through dip coating method. Accompanied with the aligned porous vessel structure of the substate materials, light from solar can be sufficiently absorbed by CNTs, achieving 98% light extinction efficiency with an evaporation rate of to 1.50 kg m^−2^ h^−1^ (Figure 6C, right).

Carbon black (CB) as the photothermal materials has been widely proved to be outstanding like plasmonic nanometals. Notably, the cost of CB is lowest, allowing to scale-up CB-based evaporator. Incorporating CB into cellulose aerogel has been demonstrated to be used for SVG system. With the different concentration of CB, the evaporator showed different color and light absorption abilities. With ingenious design, the system reached 112% efficiency under 0.2-sun.

### Polymer Materials

Even though the options are limited, polymer-based photothermal materials have a wide range of applications in SVG because of their flexibility which enable efficient deposition on various substrates and outstanding photothermal conversion ability due to lattice vibrations. Polypyrrole (PPy), polyanion (PANI), polydopamine (PDA) are the most well studied photothermal polymers for solar evaporation (Cao et al., 2019). All of them could be *in situ* polymerized on different substrates, including cellulose, wood, metal, and other polymers.

PDA with active functionals groups (catechol, amine, imine) can adhere on the substrate by multiple hydrogen bonding. The evaporator exists when PDA cooperated with fibrous aerogels (e.g., cellulose), in which PDA was *in-situ* polymerized and coated on the surface of the fibrous network. The resulted PDA-incorporated aerogel achieved light extinction over 90% (300–2,500 nm) (Zou et al., 2021). The functional groups of PDA that could not only serve as anchors for efficient organic contaminant removal, but also endows materials with hydrophilicity. Therefore, the PDA/cellulose aerogel was demonstrated to be used for water remediation through both evaporation and adsorption towards multi-contaminated seawater with organic dyes or oil spillage. The metal ion concentration and organic contents of condensed water were qualified for drinking standards defined by WHO.

PPy, similar to carbonaceous materials, has strong light absorption capabilities and can stably polymerized on the substrate. Alkali-treated corn straw is an example to load PPy as solar absorber (Li J. et al., 2020). PPy was coated on the surface of microchannel of corn straw, forming a black appearance of evaporator. The evaporation rate of the resulted evaporator reached 1.67 kg m^−2^ h^−1^ with 96.8% energy conversion efficiency under one-sun illumination. Meanwhile, the hydrophilic nature of PPy ensured the good salt resistance.

### AIE Materials

A well-tailored aggregation-induced emission (AIE)-active molecules as newly emerging photothermal agents could be a promising candidate for SVG. Generally, AIE refers to a unique phenomenon in which the energy of excited state decays by non-radiative pathways in dilute solution, while in the aggregated state, the intramolecular motions are restricted and the energy decays primarily through radiative pathway, resulting in strong fluorescent emission in the aggregated state (Hong et al., 2011; Chen Y. et al., 2019). In the tailored structure of AIE luminogen, the molecules can retain the motion and the excited-state energy can dissipate through non-radiative decay route, generating heat during the process, which can be used as photothermal material (Figure 6D, left). The all-fiber aerogel for SVG using photothermal AIE molecules and nanofibers was fabricated (Li et al., 2020a). The AIE molecule MTTT-BT chiefly absorbs light between 300 and 750 nm, the range which contributes the most energy to solar spectrum. AIE molecule was dissolved with poly(vinylidene fluoride-co-hexafluoropropylene) (PVDF-HFP) solution and electrospun to build 2D nanofibrous mat, which was subsequently blended to achieve uniform dispersion. The all-fiber aerogel containing the AIE molecule was prepared after freeze-drying (Figure 6D, right). The 3D aerogel possessed outstanding properties such as interconnected porous network, excellent water transportation and exceptional light-to-heat efficiency (1.43 kg m^−2^ h^−1^ with 89% photothermal efficiency). Additionally, through rational design, the AIE molecule could simultaneously be used as high performance photothermal material as well as reactive oxygen species generator, making it a promising choice for SVG and antibiofouling function. The 3D fibrous aerogel using nanofiber and a typical D-A-D AIE molecule (TPA-BTDH) with this proposal was prepared (Li H. et al., 2021). The photosensitization of TPA-BTDH was studied, and the emission intensity of reactive oxygen species (ROS) indicator was significantly boosted, demonstrating the efficiency of reactive oxygen species generation. The 3D evaporator was constructed by expanding the 2D nanofibrous mat, which provided the evaporator with porous structure and superior water absorption capability. The development of a 3D evaporator with excellent evaporation properties (3.6 kg m^−2^ h^−1^) and antibiofouling performance enabled the construction of the next generation evaporator for water purification.

The summary of the materials and performance of the SVG system based on different absorbers is reported in Table 1. The challenges of photothermal materials locate on the stability and scalability, which need further exploration.

**TABLE 1:** SVG performances of different photothermal materials and fibrous building blocks.

Photothermal materials	Building blocks	Absorption (%)	Evaporative rate (kg m^−2^ h^−1^)	Efficiency (%)	References
Au nanorods	CNF		11.52 (5 sun)	76.3	Tian et al. (2016)
CuS nanoparticles	BNC	97.9 (250–2,100 nm)	1.44 (1 sun)	83.5	Zhang et al. (2021a)
MoS_2_ nanosheets	BNC	96 (450–750 nm)	6.15 (5.35 sun)	81.4	Ghim et al. (2018)
rGO	BNC	96 (400–1,100 nm)	11.8 (10 sun)	83	Jiang et al. (2016)
rGO	Rice-straw-fibers	96 (290–1,400 nm)	2.25 (1sun)	88.9	Storer et al. (2020)
CNTs	CNF	97.5 (300–1,200 nm)	3.52 (3 sun)	81.4	Jiang et al. (2018)
CNTs	SiO_2_ nanofibers	98 (200–2,500 nm)	1.50 (1 sun)	85.4	Dong et al. (2020)
CB	CNF	97.5 (250–2,500 nm)	1.12 (outdoor)	89.1	Liu et al. (2019b)
PDA	CNF	90 (300–2,500 nm)	1.36 (1 sun)	86	Zou et al. (2021)
PPy	corn straw fibers	95 (200–2,500 nm)	1.67 (1 sun)	96.8	Li et al. (2020c)
PEDOT-PSS	CNF	99 (250–2,500 nm)	1.61 (sun)	81	Han et al. (2020)
MXene	CNF	95.8 (200–2,500 nm)	2.287 (1sun)	88.2	Han et al. (2021)
AIE molecules	PVDF-HFP nanofibers	90 (250–2,500 nm)	1.43 (1sun)	89	Li et al. (2020a)
AIE molecules	PMMA nanofibers	80 (250–2,500 nm)	3.6 (1sun)		Li et al. (2021a)

Based on optimized design and synthesis of fibrous building block and photothermal materials, the basic ingredients of evaporator have been obtained. In next part, how to assemble them with the anticipated structure will be illustrated.

## Pore Structures Construction

In order to enhance the water transportation and light absorption as well as lower the thermal conductivity of evaporator, the porous structure of evaporator is important. The porous structure of solar evaporators could be constructed in various ways based on fibrous materials mentioned above. In this section, we will discuss the design guideline for porous structures as well as the methods to achieve it.

Evaporator with porous structure is advantageous for thermal management due to their low thermal conductivity and fast water transportation via capillary effect (Jiang et al., 2018). Moreover, the light absorption can be enhanced by multiple reflection in the porous structure. Nonetheless, 3D fibrous aerogels usually show irregular and disordered porous structures, on the other hand, exhibit lower evaporation rates, which could be ascribed to the resistance during water transportation process (Zhang Q. et al., 2019).

Hence, inspired by natural plant transpiration, the methods to prepare similar porous structures are attractive, wherein water transports in the vertical aligned channels of the tree trunks with low flow resistance (Tu et al., 2020). The porous structures of fibrous aerogels could be prepared by different methods, including plant templating, ice templating, foaming, and 3D printing, considering various strategies to improve the performance of SVG systems (Zhang et al., 2005; Deville et al., 2006; Li et al., 2017c; Shao et al., 2020; Chen et al., 2021b).

**FIGURE 7:**
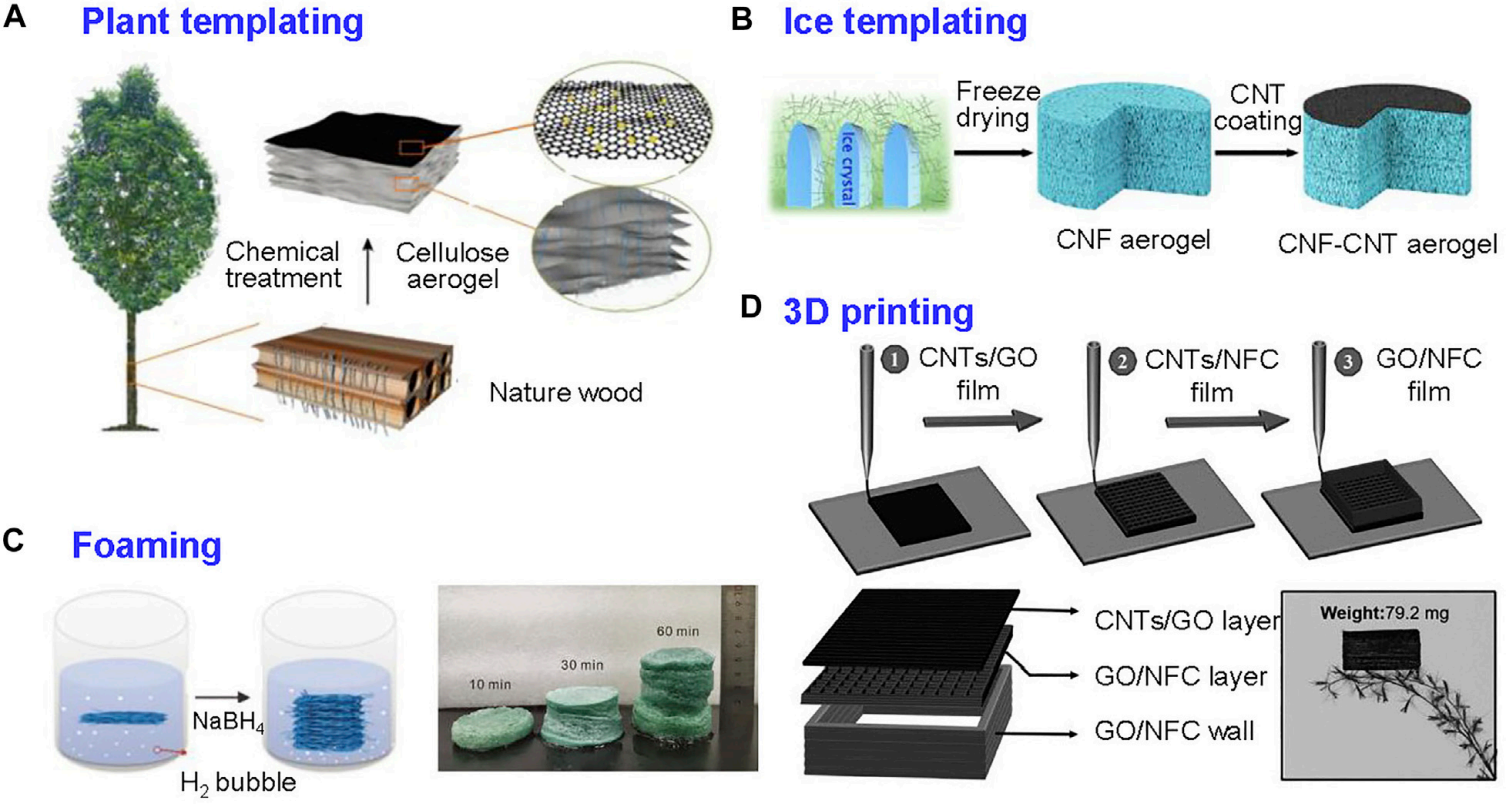
Porous structures construction strategies for fibrous aerogels. **(A)** Plant templating: utilize the origin structure of nature plant to produce cellulose aerogels. Reprinted with permission from (Zhang Q. et al., 2020). Copyright (2020) American Chemical Society. **(B)** Ice-templating: prepare fibrous aerogels with oriented pore structure. Reprinted with permission from (Jiang et al., 2018). Copyright (2018) American Chemical Society. **(C)** Foaming: produce porous fibrous aerogels with expansion treatment of 2D nanofibrous membrane. Reprinted with permission from (Li H. et al., 2021). Copyright (2021) WILEY VCH. **(D)** 3D printing: design fibrous aerogels with programmed control of ink extrusion. Reprinted with permission from (Li et al., 2017c). Copyright (2017) WILEY VCH.

Wood, corn straw, and Juncus pith are the examples of natural plants with aligned channels for efficient water transportation (Wang F. et al., 2020; Tu et al., 2020). To fully exploit the natural aligned porous structures, the plants have been chosen as templates which were chemically treated to remove excess components, and the obtained fibrous and porous structure was used as substrate for SVG system when coupled with photothermal materials (Figure 7A).

To mimic the porous structure of natural plant, ice templating is an efficient method to prepare aligned porous structure into 3D materials (Shao et al., 2020). The pore structure is imparted by introducing ice into the solution containing aforementioned materials. The pore structure is imparted by introducing ice into the solution containing aforementioned materials. During this process, the materials concentrate between the neighboring ice crystals, and then ice removed after sublimation or thawing, leaving the pore structure as the negative cast of the original ice crystals (Figure 7B). Unidirectional freezing produces uniaxial aligned ice crystals, with the pore structure orienting along a single direction, which benefits water transport and thermal insulation (Jiang et al., 2018). This method has been used to construct fibrous building blocks such as cellulose, polymer nanofibers, and inorganic nanofibers (Long et al., 2018; Qin et al., 2021; Liu et al., 2022). Ice templating is a versatile manufacturing technique, but the time and energy consuming properties of this method limits the large-scale production of materials.

Foaming is another approach to develop porous structures. A variety of chemical and physical blowing agents have been used to prepare polymer foam such as thermoplastic and thermosetting polymers (i.e., polypropylene and polyurethane) (Qiao et al., 2021). The foaming agent could react, generating a gas bubble and enter the interior gap of the structure. Nowadays, the foaming approach is used to fabricate porous foam for SVG evaporator, including nanofibers and 2D nanosheets. Electrospun nanofiber with 2D compact texture is contrary to the demand of SVG (Li H. et al., 2021). The compact fibrous mat could be expanded into 3D porous structure using gas-foaming approach (Figure 7C). The low density ensures the floating of foam on the surface of water, and the porous structure contributes low thermal conductivity to reduce thermal loss to bulk water, making it suitable for SVG systems. The foaming approach does not require post-dying process or template to construct pore structure, but the controlled manipulation should be evaluated.

3D printing is a process in which material is constructed under computer control. 3D printing to fabricate porous SVG evaporator is an ingenious method (Li et al., 2017c). Three dimensional metamaterials derived from 3D printing have gained growing popularity amid various approaches. The method known as direct ink writing is useful in printing diverse sophisticated structures using colloidal gel inks such as nanocellulose, graphene, CNTs, and polymers (Chen et al., 2021b). The ink could be directly assembled after being extruded by needle with designed structure, which relies on the rheological properties of materials. Nanocellulose is a preferable candidate used as the building block with direct ink writing because of the suitable rheological properties and interaction between adjacent extrusion fibers (Figure 7D). Benefitting from this method, various 3D printed fibrous aerogels could be tailored and constructed with sophisticated and accurate architecture for SVG applications(Koh et al., 2021).

The pore structure of fibrous aerogel is important, which is the keystone for thermal insulation and water transportation. The pore structure fabrication strategy should be carefully designed with comprehensive consideration of use scenarios and corresponding advantages.

## Device Design

The basic porous SVG evaporator is developed to integrate three components of photothermal materials, water path, and thermal insulation layer together. However, the performance of evaporator must be further improved by rational design to fulfill the requirements of highly efficient water generation (Xu et al., 2017; Zhao et al., 2020). In this section, we will discuss the design strategy based on the thermal and water management.

### Thermal Management

Energy loss is an unfavorable factor in the reduction of evaporation efficiency of SVG system, which could occur during photothermal conversion, thermal conduction to bulk water, and surface-to-environment radiation and convection (Wu et al., 2021). To increase the conversion efficiency of evaporator, the energy from solar light should be used effectively and the thermal input into the evaporator should be used to generate steam as much as possible.

Conduction, convection and radiation are the three modes of heat transfer (Wicklein et al., 2014). As the temperature of evaporator is higher than that of water and environment, heat is inevitably lost through radiation, conduction, and convection, and only the energy used for water evaporation counts as useful and determines the energy efficiency of the system (Liu Z. et al., 2017). Hence, the thermal management of SVG evaporator is critical to achieve high energy efficiency.

In the development of SVG, three strategies are used: bottom heating, bulk heating, and interfacial heating (Tao et al., 2018). The bottom heating strategy places solar absorber into the bottom of water, where the light is absorbed and then converted to thermal energy to heat the bulk water. The bulk heating strategy incorporates a uniformly dispersed solar absorber into the bulk water, then the light is converted to thermal energy to heat the liquid. The aforementioned two strategies are based on absorber-in-water principle, and the percentage of heat energy transferred to water evaporation is lower. Therefore, the water-in-absorber principle known as interfacial heating strategy, utilizes heat localized at air-liquid interface to generate vapor, and then improves the conversion finally.

**FIGURE 8:**
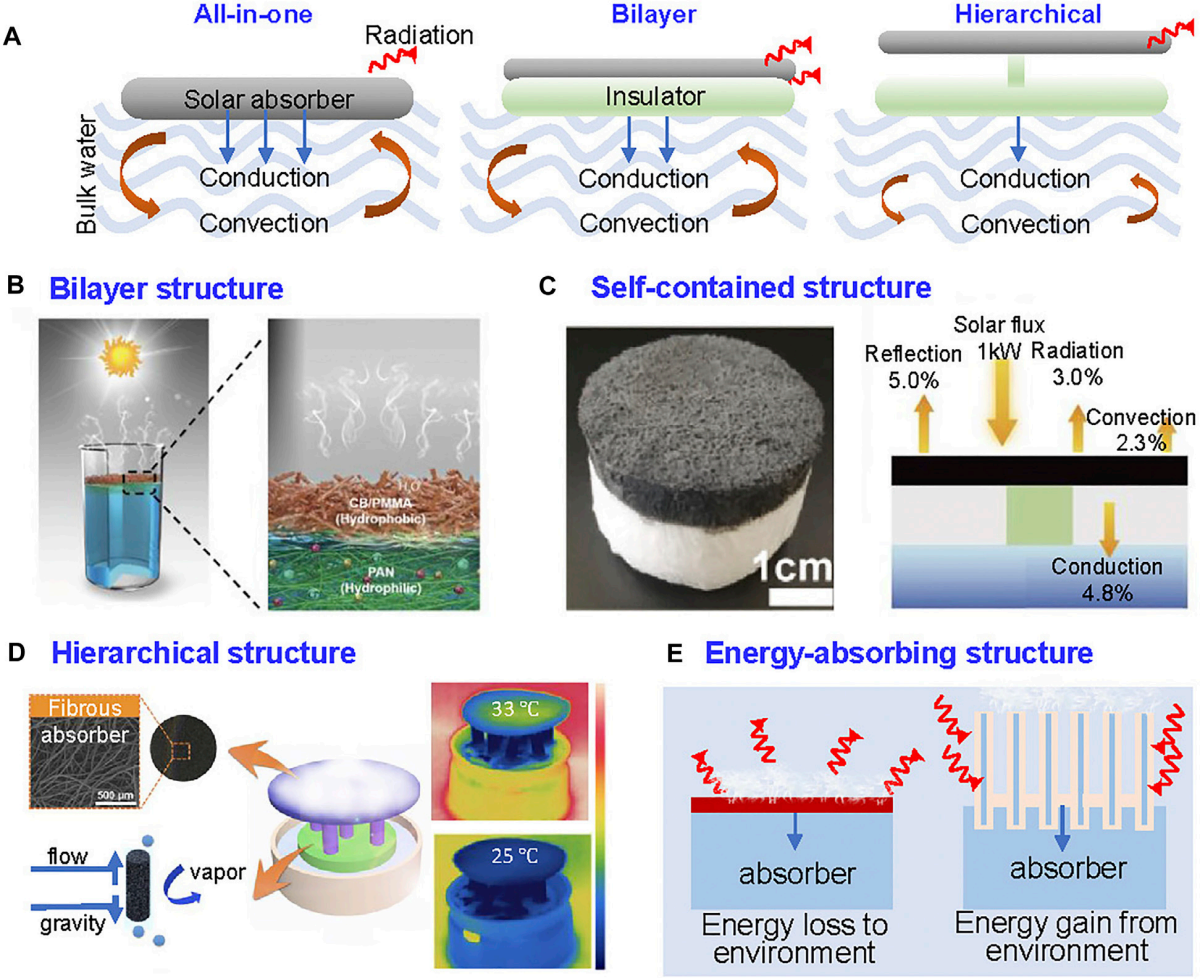
Thermal management strategies of efficiency enhancement of SVG. **(A)** Working principles of all-in-one, bilayer, and hierarchical structures of SVG system (Zhao et al., 2020). **(B)** Bilayer fibrous aerogels with top hydrophilic layer and bottom hydrophobic layer. Reprinted with permission from (Xu et al., 2018). Copyright (2018) WILEY VCH. **(C)** Structure of the self-contained fibrous aerogel and its operation principle. Reprinted with permission from (Li N. et al., 2021). Copyright (2020) WILEY VCH. **(D)** Structure of the hierarchical fibrous aerogel and its working performance. Reprinted with permission from (Yu Z. et al., 2019). Copyright (2018) American Chemical Society. **(E)** Working principles of the energy-absorbing SVG device by extending evaporator length (Li et al., 2018).

The most common fibrous aerogels design for interfacial heating can be divided into three parts: all-in-one design, bilayer design and hierarchical structure (Figure 8A; Zhao et al., 2020). The photothermal material is uniformly dispersed in the aerogel that belongs to all-in-one design. In this case, heating energy could penetrate through the whole porous structure via conduction, causing inevitable conduction loss. To address this problem, the bilayer structured SVG system came into being, which refers to the fact that the photothermal material is deposited on the surface of system, with the insulator underneath. The water can be pumped to the surface of air-liquid surface through porous structure with capillary effect using hydrophilic fibrous network. The thermal energy confines on the surface of floating aerogel (upper) rather than the entire body of evaporator, localizing the heat. The energy loss through aerogel to bulk water is less, as a result, more energy can be allocated for water evaporation (Figure 8B). The overall conversion efficiency has been improved to over 90% with the 3D metamaterials (Li N. et al., 2021).

As thermal energy could still conduct through the hydrophilic layer, a self-contained structure design with a hydrophobic layer as the bottom layer and hydrophilic layer as the top layer with water transportation via water channel has been developed (Figure 8C; Yu Z. et al., 2019; Wu et al., 2019). The hierarchical structure of top and bottom layers could connect through water channels, further restricting thermal loss through porous structure (Figure 8D). With such design, the heat energy could be further localized in the air-liquid surface thereby eliminating energy loss to bulk water. Only the problem of this design is that requires multi-step preparation.

Although great efforts have been made to minimize the thermal energy loss to environment, the theoretical limit of 100% energy utilization for vapor generation stymies further development. Because the temperature of evaporator is higher than ambient temperature, the heat transfer to environment through radiation is inevitable. To overcome the limitation, the temperature of side aera of evaporator was controlled to lower than environment with the rational design to absorb environment energy (Li et al., 2018; Wu et al., 2021). Through extending the length of the device, the majority of the system is exposed to the environment. Therefore, energy is gained from the environment because the absorbers are lower in temperature than the surrounding environment, yielding an evaporation rate over 100% that exceeds the theoretical value (Figure 8E).

### Water Management

**FIGURE 9:**
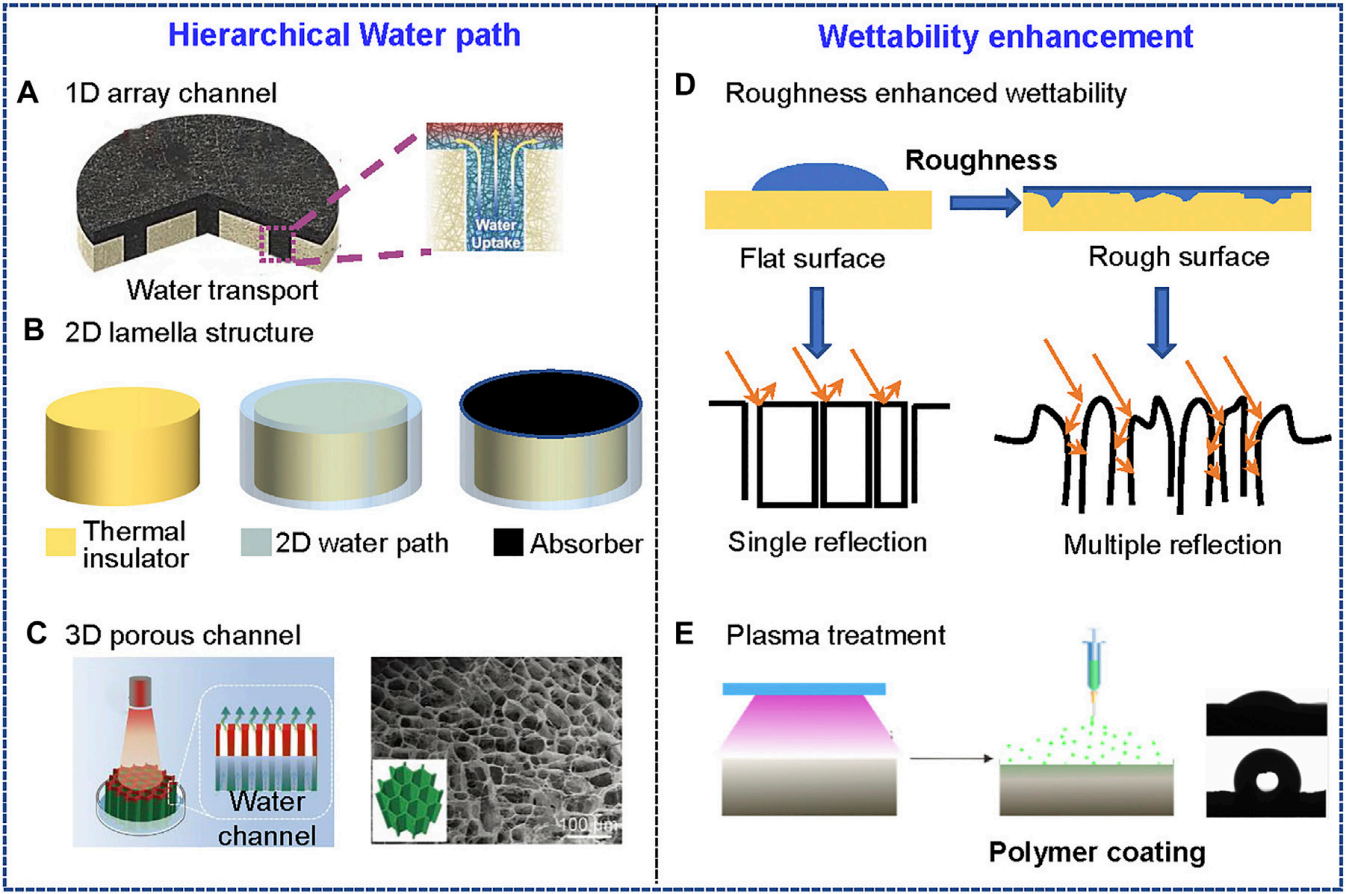
Water management strategies for efficiency enhancement of SVG. **(A)** Water supply which is delivered by 1D array channel with reduced contact aera to minimize conduction heat loss. Reprinted with permission from (Gao T. et al., 2019). Copyright (2019) WILEY VCH. **(B)** Water supply enabled by 2D lamella structure that is integrated with thermal insulator underneath the water path (Li et al., 2016). **(C)** Water supply provided by 3D porous channel that is directly soaking in water. Reprinted with permission from (Xiong et al., 2021). Copyright (2021) WILEY VCH. **(D)** (top) Enhanced wettability provided by rough surface (Drelich et al., 2011). (bottom) The light absorption is increased by multiple reflection on rough surface when compared to that of flat surface (Qiu et al., 2019). **(E)** Plasma treatment for fiber surface hydrophilicity modification. The water contact angle measurement exhibited the treated surface and untreated surface with the opposite wettability on two sides of aerogel. Reprinted with permission from (Li et al., 2020a). Copyright (2020) American Chemical Society.

Water management, like thermal management, is crucial in the evaporation process (Zhao et al., 2018; Xu et al., 2019). Isolating the evaporation surface could reduce heat conduction from evaporator to the water by interfacial heating strategy. During the process, adequate water supply is critical for improving the evaporator performance and achieving higher efficiency, the pathway used to transport water differs among evaporators(Zhang Y. et al., 2020). The water pathways can be divided into three categories on the basis of thermal management design: 1D array, 2D lamella, and 3D porous structure (Li et al., 2016; Li et al., 2017b; Xiong et al., 2021; Figures 9A–C). The 1D array is a narrow path for water to climb up to the surface of system, mainly intended to reduce heat loss. The design of 1D array is inspired by nature materials, such as mushroom and tree stem (Si et al., 2014). The evaporation area absorbs water and generates vapor under solar energy within directed array for water to transport from bottom to top. The problem may arise in this design when the evaporation area is too large for sufficient water supply through it during the evaporation process (Figure 9A). The structure of 2D lamella path for water transport is achieved by coating a hydrophilic layer onto a thermal insulator, which pumping water by capillary force. The capillary effect posed by lamella material determines the efficiency of system (Figure 9B). The fibrous aerogels with aligned channels are favorable for water transport, as water could be efficiently pumped to the surface of evaporator through porous structure based on strong capillary effect (Figure 9C). Nonetheless, the conduction loss through the 3D water path is inevitable. Therefore, combination of these three designs in smart way is a promising strategy to balance the water transport and thermal insulation.

Integrate the 1D array and 3D porous structure, the fibrous aerogel can transport water through the porous water channel that wrapped by thermal insulating floating layer, significantly enhanced the water supply and reduced heat loss (Li N. et al., 2021). As mentioned above in Figure 3E, the water path was the CNF aerogel crosslinked with glutaraldehyde to ensure mechanical stability and hydrophilicity. In order to float on the water to limit energy loss to bulk water, the thickness of hydrophobic layer was rationally selected. The water pathway in this design could sufficiently pump water to the solar absorber layer with minimum energy loss to fluid.

### Wettability Enhancement

The wettability of solar absorber layer influences the speed of water spreading at interfaces and the absorption of solar light.

According to the contact angle equation described by Wenzel (Wenzel, 1936) for complete wetting condition:

$$\cos\theta_{r}^{w}=r\cos\theta_{e}$$

Where $\theta_{e}$ is the effective contact angle on the rough surface, and *r* is the factor of roughness. When the fiber is hydrophilic and rough, $\theta_{e}<90{}^{\circ}$ , the effective contact angle would be small enough, implying that the surface could be superhydrophilic and promotes water spread (Qiu et al., 2019).

As the air could be trapped in the space between nanofibers, the Wenzel’s model is not proper for contact angel, and Cassie-Baxter’s model is more suitable for this situation with contact angle can be predicated, which refers to a wetting state where a liquid drop sits on top of a surface’s rough features and traps pockets of gas between these features (Michielsen and Lee, 2007; Shardt and Elliott, 2018):

$$\cos\theta_{r}^{CB}=r_{f}f\cos\theta_{e}+f-1$$

where f is the fraction of the projected area of the solid surface in contact with the liquid and $r_{f}$ is the roughness of the portion of the solid that is in contact with water. When f=1 , the CB model turns to the Wenzel’s model. For nanofiber with hydrophobicity ( $\theta_{e}>90{}^{\circ}$ ), the fraction projected area (*f*) of the solid surface in contact with the liquid of fibrous aerogel is significantly less than 1, resulting the superhydrophobic surface (Michielsen and Lee, 2007). For hydrophilic nanofiber, the groove cannot completely prevent water penetration of nanofiber. Hence, the fraction of contact aera is close to 1 and the $\theta_{r}^{CB}$ can be small enough. Therefore, with the contact angle of fiber less than 90^o^, the wetting phenomenon of fibrous aerogel could be completely wetting as the $\theta_{r}^{CB}$ is near to 0^o^, which is beneficial for water spreading and vapor emission (Zhang et al., 2017).

The speed of redistribution of water is critical for SVG process. The fibrous aerogels produced by ice templating or foaming would have enough micro-nano structure with superhydrophilic property, and light absorption of fibrous structure could be further enhanced in comparison to flat surface, contributing to a better SVG performance (Figure 9D).

Some fibrous building blocks are hydrophobic, hydrophilic treatment is required for water transport. Oxygen plasma is a facile method to introduce hydrophilic oxygen-contained groups onto substrate (Jia et al., 2020). For example, the poly(vinylidene fluoride-co-hexafluoropropylene) (PVDF-HFP) nanofiber is hydrophobic, after being treated with oxygen plasma on one side, the hydrophobic layer turned to be hydrophilic (Li et al., 2020a). Then, the hydrophilic polymer gelatin nanoparticles were deposited on the treated side by electro-spraying. With enough exposure time to spray and cross-linking via glutaraldehyde vapor, the fibrous aerogel was permanently turned hydrophilic (Figure 9E). The fibrous aerogel has two opposing wettability levels, which could be used to control the direction of water transport.

## Conclusion and Perspective

This review provided an overview of the 3D fibrous aerogels used for solar vapor generation. Various fibrous building blocks based on natural cellulose nanofibers, petrochemical polymer nanofibers, and inorganic nanofibers are emerging to fabricate aerogels. Photothermal materials such as plasmonic nanometals, semiconductors, carbonaceous polymers, MXene and AIE molecules have been explored to combine with fibrous substrate for solar-to-heat conversion. The porous structures are crucial in the fabrication process when using the proper method to assemble both fibrous building blocks and photothermal materials. Great improvements in evaporation efficiency, including thermal and water management, have been made with a suitable SVG strategy. The interfacial evaporation could limit the heat loss to the bulk water based on energy confinement. Water management for efficient SVG could be optimized through coupling different water paths and processing the wettability of the evaporator. Compared to other technologies used for water purification, the SVG-based technology is promising for both large-scale as well as personal use with inexhaustible solar energy and independent of electricity and complex infrastructure.

Having various materials and structural designs that have been successfully applied to SVG, the optimized design is still required for further improving the efficiency and practical application. First, the hydrophilicity of the fibrous building blocks could be further tailored to increase the interaction with bonded water, then weakening the hydrogen bonds of the intermediate water to reduce the evaporation enthalpy of water, and ultimately improve the SVG efficiency. Second, multifunctional SVG with antibiofouling activity, photocatalysis, energy transformation property is the trend of the next generation evaporator. Finally, simple fabrication methods and low-cost starting fiber materials are still needed for large-scale SVG production, allowing them for practical application to alleviate the water scarcity and facilitate the sustainable development.
